# Toll-like receptors genes polymorphisms and the occurrence of HCMV infection among pregnant women

**DOI:** 10.1186/s12985-017-0730-8

**Published:** 2017-03-24

**Authors:** Wioletta Wujcicka, Edyta Paradowska, Mirosława Studzińska, Jan Wilczyński, Dorota Nowakowska

**Affiliations:** 1 Scientific Laboratory of the Center of Medical Laboratory Diagnostics and Screening, Polish Mother’s Memorial Hospital—Research Institute, 281/289 Rzgowska Street, Lodz, 93-338 Poland; 2 Department of Perinatology and Gynecology, Polish Mother’s Memorial Hospital—Research Institute, 281/289 Rzgowska Street, Lodz, 93-338 Poland; 3 2nd Chair of Obstetrics and Gynecology, Duchess Anna Mazowiecka Public Teaching Hospital, Warsaw, Poland; 4 Laboratory of Molecular Virology and Biological Chemistry, Institute of Medical Biology, Polish Academy of Sciences, Lodz, Poland

**Keywords:** Human cytomegalovirus (HCMV), Toll-like receptors (TLRs), Single nucleotide polymorphism (SNP), Infection, Pregnant women

## Abstract

**Background:**

Human cytomegalovirus (HCMV) is the most common cause of intrauterine infections worldwide. The toll-like receptors (TLRs) have been reported as important factors in immune response against HCMV. Particularly, TLR2, TLR4 and TLR9 have been shown to be involved in antiviral immunity. Evaluation of the role of single nucleotide polymorphisms (SNPs), located within *TLR2*, *TLR4* and *TLR9* genes, in the development of human cytomegalovirus (HCMV) infection in pregnant women and their fetuses and neonates, was performed.

**Methods:**

The study was performed for 131 pregnant women, including 66 patients infected with HCMV during pregnancy, and 65 age-matched control pregnant individuals. The patients were selected to the study, based on serological status of anti-HCMV IgG and IgM antibodies and on the presence of viral DNA in their body fluids. Genotypes in *TLR2* 2258 A > G, *TLR4* 896 G > A and 1196 C > T and *TLR9* 2848 G > A SNPs were determined by self-designed nested PCR-RFLP assays. Randomly selected PCR products, representative for distinct genotypes in *TLR* SNPs, were confirmed by sequencing. A relationship between the genotypes, alleles, haplotypes and multiple variants in the studied polymorphisms, and the occurrence of HCMV infection in pregnant women and their offsprings, was determined, using a logistic regression model.

**Results:**

Genotypes in all the analyzed polymorphisms preserved the Hardy-Weinberg equilibrium in pregnant women, both infected and uninfected with HCMV (*P* > 0.050). GG homozygotic and GA heterozygotic status in *TLR9* 2848 G > A SNP decreased significantly the occurrence of HCMV infection (OR 0.44 95% CI 0.21–0.94 in the dominant model, *P* ≤ 0.050). The G allele in *TLR9* SNP was significantly more frequent among the uninfected pregnant women than among the infected ones (χ^2^ = 4.14, *P* ≤ 0.050). Considering other polymorphisms, similar frequencies of distinct genotypes, haplotypes and multiple-SNP variants were observed between the studied groups of patients.

**Conclusions:**

*TLR9* 2848 G > A SNP may be associated with HCMV infection in pregnant women.

**Electronic supplementary material:**

The online version of this article (doi:10.1186/s12985-017-0730-8) contains supplementary material, which is available to authorized users.

## Background

Human cytomegalovirus (HCMV) is the most common cause of intrauterine infections worldwide, with seroprevalence rates at the range from 40 to 100% among pregnant women [1–4]. Our recent study, performed among Polish pregnant women between 2010 and 2011, showed seroprevalence of anti-HCMV IgG and IgM antibodies to have been 62.4 and 2.2%, respectively [4]. Compared to other European populations of pregnant women from the Netherlands (41%), France (46%) and the United Kingdom (49%), the prevalence of IgG anti-HCMV in the Polish pregnancy cohort was still high [5–7]. In case of primary infections, diagnosed during pregnancy, the transplacental transmissions of the virus from mother to fetus occur with the incidence rate of 30–40%, while the recurrent infections cause congenital cytomegaly within the range 0.2–2.2% [8–12]. Among fetuses and neonates, congenitally infected with HCMV, cytomegaly may have both asymptomatic and symptomatic course with severe symptoms, including microcephaly, ventriculomegaly, increased periventricular echogenicity and calcifications [8, 12].

Taking into account the immune response to HCMV, the Toll-like receptors (TLRs) have been reported to play important role [13–15]. Particularly, TLR2, TLR4 and TLR9 have been shown to be involved in antiviral immunity [14, 16–18]. In the most recent *in vitro* study, TLR2 was found as a target of HCMV miR-UL112-3p [19]. Previously, TLR2 was also determined to be involved in the functional sensing of HCMV through direct interactions with viral glycoproteins (gp, g) gB and gH [20]. In turn, TLR4 was reported to be correlated with inhibition of HCMV infection [21]. In human monocytoid THP1 cells and foreskin fibroblasts, TLR9 was determined to induce the expression of TNF-α at 1 h after HCMV infection [22]. A study performed for neonatal human fibroblasts, showed some involvement of TLR9 in the development of HCMV infection as well [23].

Previously, the role of single nucleotide polymorphisms (SNPs, variations of single nucleotides at specific positions in sequences of the genes), residing within *TLR* genes, was also reported [24–26]. In case of *TLR2* + 1350 T > C polymorphic site, the CC genotype (homozygotic status with two minor C alleles) was correlated with congenital HCMV infection [27]. In turn, our recent study showed no genotypic variability within *TLR2* + 1350 T > C as well as 2029 C > T SNPs among the analyzed Polish fetuses and neonates, who were both congenitally infected and uninfected with HCMV [28]. However, the study reported the GA heterozygotic status and A allele located within *TLR2* 2258 G > A SNP to be significantly more frequent among the infected offsprings than among uninfected individuals [28]. In an in vitro study of the transfected human embryonic kidney (HEK) 293 cells exposed to HCMV gB, the *TLR2* 2258 SNP was shown to be associated with TLR2 signaling impairment [25]. Considering *TLR4*, both 896 A > G and 1196 C > T polymorphisms were reported to impair TLR4/MD2 dimerization necessary to activate downstream signaling, involved in HCMV-induced immune response [29, 30]. *TLR4* 896 A > G and *TLR4* 1196 C > T SNPs were also determined to be significantly associated with more frequent opportunistic infections and cytomegaly, diagnosed among renal transplant recipients (RTRs) [14, 31]. Another study performed for RTRs and their unrelated donors, showed both *TLR4* SNPs to be possibly associated with the risk factors of invasive aspergillosis that included HCMV seropositivity [24]. Considering *TLR9*, the -1237 T > C SNP was marginally correlated with recurrent urinary infections in RTRs [32]. The study performed for Polish fetuses and neonates congenitally infected with HCMV, showed the CC genotype in *TLR4* 1196 polymorphism, as well as the GA variant in *TLR9* 2848 G > A SNP to be correlated with the infection, and the heterozygotic status in *TLR9* SNP increased the risk of congenital cytomegaly by 4.81 times [18]. Moreover, complex AA variants for both *TLR2* 2258 and *TLR9* 2848 G > A polymorphisms, were found to be associated with an increased risk of congenital HCMV infection [28]. Regarding *TLR9* -1486 T > C and 2848 C > T SNPs, the heterozygous and homozygous recessive genotypes within the reported polymorphisms, were associated with an increased risk of HCMV disease among infants [33].

**Fig. 1:**
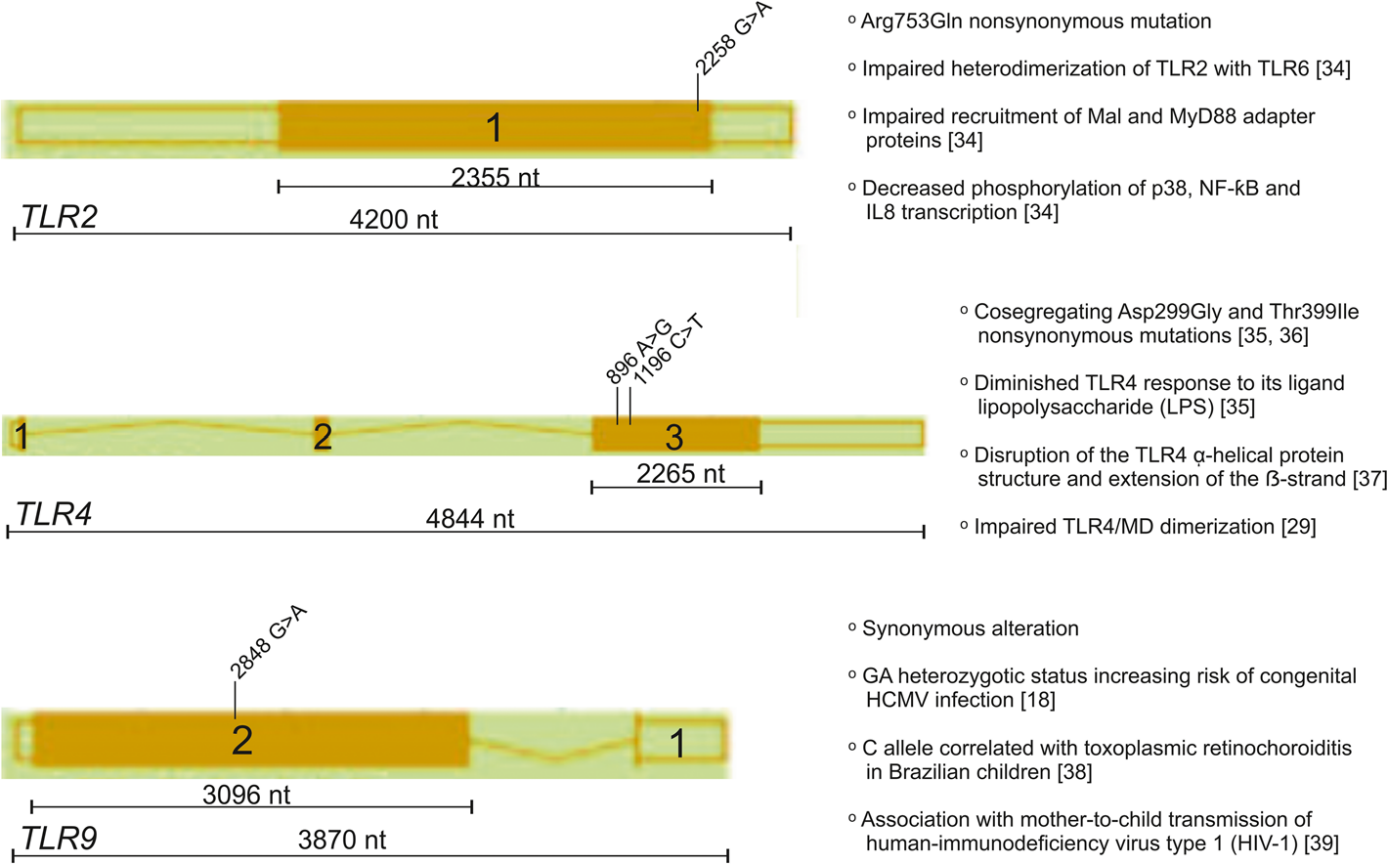
The primary structures of *TLR2*, *TLR4* and *TLR9* genes, loci of the polymorphic sites analyzed in the study, and role of the polymorphisms in immune response. Structures of the genes were developed using the Ensembl genome browser and NCBI dbSNP database. Numbered squares represent the following exons in the genes. Lengths of the exons containing analyzed polymorphisms, and of the whole genes, are shown by numbers of nucleotides (nt) over black lines. Diagonal subtitles show the names of studied SNPs. Role of the polymorphisms is indicated on the right side of genes

Considering reported data, the association between the presence of genetic changes within *TLR2* 2258 G > A, *TLR4* 896 A > G and *TLR4* 1196 C > T, as well as *TLR9* 2848 G > A SNPs and the occurrence of HCMV infection among pregnant women seems to be really possible, although there have been no such reports. Therefore, the current paper was aimed to describe the role of *TLR2*, *TLR4* and *TLR9* SNPs (see Fig. 1) in the occurrence of HCMV infection among pregnant women, acquired within the gestation period [18, 29, 34–39]. The estimation of the possible relationship between the genetic status within the analyzed polymorphic sites and the appearance of HCMV infection might be significant to provide new genetic alterations possibly associated with the infection occurring among pregnant women during pregnancy.

## Methods

The study was performed with 131 pregnant women, including 66 patients infected with HCMV during pregnancy, and 65 age-matched control individuals uninfected with the virus, at the age between 18 and 41 years (mean 28.52 years). The mean age among HCMV-infected pregnant women was 28.29 ± 5.29 years, and among uninfected pregnant women was 28.75 ± 4.79 years. The samples were obtained from pregnant women, admitted to the Department of Feto-Maternal Medicine and Gynecology at the Polish Mother’s Memorial Hospital—Research Institute, between the years 2002 and 2014. Clinical samples, used in the *TLRs*’ SNPs genotyping, consisted of whole blood and serum specimens. HCMV infection, that occurred within the pregnancy period in pregnant women, was determined by serological status for anti-HCMV antibodies, HCMV DNA detection, as well as by ultrasound markers related to congenital cytomegaly in their fetuses. Symptoms associated with congenital disease included microcephaly (determined in 1 of 66 tested fetuses and neonates, 1.52%), ventriculomegaly (1/66, 1.52%), respiratory failure (5/66, 7.58%), heart defects (4/66, 6.06%), hepatitis (1/66, 1.52%), ascites (2/66, 3.03%), intrauterine growth restriction (IUGR, 6/66, 9.09%), as well as fetal/neonatal death (7/66, 10.61%), and were observed among the 28.8% (19/66) offsprings of the infected pregnant women. Active HCMV infections were confirmed in 15 pregnant women (15/66, 22.7%) and their 10 offsprings (10/66, 15.2%), based on the presence of the viral DNA in body fluids, including maternal whole blood, plasma, and urine samples, as well as fetal amniotic fluids and umbilical cord blood specimens. Considering all the studied parameters related to HCMV infection, the overall rate of congenital viral transmission from mother to the fetus was 33.3% (22/66). The study was approved by the Research Ethics Committee at the Polish Mother’s Memorial Hospital—Research Institute. All the samples, previously collected for diagnostic purposes, were anonymized in this report. Informed consent forms were signed by all the enrolled pregnant women.

### Serological tests

Blood specimens were gained from the pregnant women by venipuncture on their first visit to the Hospital, between the 5th and 38th week of gestation (mean 21.96 weeks). The mean gestational age among HCMV-infected pregnant women was 22.50 ± 9.21 weeks, and among uninfected pregnant women was 21.63 ± 9.02 weeks. Serum samples were obtained by centrifugation and then stored at 4^o^C until analysis, on the day of blood collection. Serological status was determined at the Hospital’s Department of Clinical Microbiology.

Screening for anti-HCMV IgG and IgM antibodies was performed with VIDAS CMV IgG and IgM tests (bioMérieux, France), used between the years 2002 and 2006. Both IgG and IgM antibodies titers ≥ 6 IU/ml were determined as positive. Between 2006 and 2011, the antibodies levels and IgG avidity, were evaluated by the chemiluminescence immunoassays (CLIA), using anti-CMV IgG and IgM tests (Diasorin/Biomedica, Italy). Samples were considered as IgG- or IgM-positive for antibody levels ≥ 0.6 IU/ml and ≥ 30 IU/ml, respectively. The IgG avidity with indexes < 0.200 was interpreted as low, 0.200–0.300 as borderline, and ≥ 0.300 as high. From the year 2012, the CLIA method was replaced by the enzyme-linked fluorescence assays (ELFA), used to determine antibodies levels. IgG antibody titers ≥ 6 IU/ml and IgM indexes ≥ 0.9 were considered positive.

### DNA extraction

Genomic and viral DNA was extracted from studied body fluids, using a QIAamp DNA Mini Kit (QIAGEN, Hilden, Germany). The isolated DNA was diluted in 100 μL of elution buffer and stored at -20^o^C until further genetic analyses, according to the manufacturer’s guidelines.

### Quantification of HCMV DNA

Among HCMV infected patients, the amount of viral DNA in the study specimens, was determined by the quantitative real-time PCR assay for the detection of *UL55* gene fragment, as described previously [40–42]. The reactions were performed in triplicate and the PCR conditions were as follows: initial activation for 10 min at 95°C and 50 cycles of repeated denaturation at 95°C for 15 s and annealing at 60°C for 1 min. The standard curves were obtained from serial 10-fold dilutions from 10^5^ to 1 plasmid DNA, containing the entire HCMV *UL55* open reading frame [42, 43]. The amplification was performed by a 7900HT Fast Real-Time PCR System (Applied Biosystems, USA).

### Genotyping of *TLR2*, *TLR4* and *TLR9* SNPs

*TLR2* 2258 G > A, *TLR4* 896 A > G and 1196 C > T SNPs, as well as *TLR9* 2848 G > A SNP (see Fig. 1), were genotyped, using the self-designed nested PCR-RFLP assays. The sequences of the external and internal primers, the lengths of PCR products and annealing temperatures, used in the PCR assays for *TLR* SNPs, were previously shown [18, 28, 44]. The sequences, annealing temperatures, and the sizes of amplicons for *TLR* SNPs are presented in Table 1. The sequences of external primers were developed, using the Vector NTI Suite 5.5 software, whereas the internal primers were taken from the literature [45–49]. Nested PCR products were digested overnight with AciI, NcoI, HinfI or BstUI endonucleases, in order to study the genotypes residing within the analyzed *TLR2* 2258 G > A, *TLR4* 896 A > G, *TLR4* 1196 C > T or *TLR9* 2848 G > A SNPs, respectively. The genotypes and alleles were determined by the length of restriction fragments, resolved on 2% agarose gels, as previously described [45–49]; see Additional file 1: Figure S1. Randomly selected amplicons for the studied *TLR* SNPs were then sequenced by the Sanger method, at the Genomed Joint-Stock Company (Warsaw, Poland), to verify the genotypes, determined by the PCR-RFLP assay. Sequencing was performed for five GG homozygotes and seven GA heterozygotes in *TLR2* SNP, for six AA homozygotes and four AG heterozygotes in *TLR4* 896 A > G SNP, for five CC homozygotes and five CT heterozygotes in *TLR4* 1196 C > T SNP, as well as for five GG homozygotes, five GA heterozygotes and five AA homozygotes in *TLR9* SNP. The exemplary chromatograms for DNA fragments of sequenced PCR products encompassing different *TLR* SNPs are presented in Additional file 2: Figure S2. In order to determine the analyzed genotypes, the sequenced and the reference fragments of the analyzed *TLR* genes were compared, using the BLASTN program for the alignment of two (or more) sequences (http://blast.ncbi.nlm.nih.gov/Blast.cgi?PAGE_TYPE=BlastSearch&BLAST_SPEC=blast2seq&LINK_LOC=align2seq). Chromatograms were read, using the Chromas Lite 2.1.1 program.

**Table 1:** Primer sequences, annealing temperatures and amplicon lengths, obtained in nested PCR assays for SNPs in the *TLR* genes

Gene	GenBank Accession No.^a^	SNP^b^ name	Primer sequences (5’-3’)		Annealing temperature [^o^C]	Amplicon length (bps) ^c^
*TLR2*	NC_000004.12	2258 G > A (rs5743708)	External	For: CGGAATGTCACAGGACAGC Rev: GGACTTTATCGCAGCTCTCAG	52	605
			Internal	For: GCCTACTGGGTGGAGAACCT Rev: GGCCACTCCAGGTAGGTCTT	59	340
*TLR4*	NG_011475	896 A > G	External	For: AAAACTTGTATTCAAGGTCTGGC	52	355
		(rs4986790)		Rev: TGTTGGAAGTGAAAGTAAGCCT		
			Internal	For: AGCATACTTAGACTACTACCTCCATG	61	188
				Rev: AGAAGATTTGAGTTTCAATGTGGG		
		1196 C > T	External	For: AGTTGATCTACCAAGCCTTGAGT	52	510
		(rs4986791)		Rev: GGAAACGTATCCAATGAAAAGA		
			Internal	For: GGTTGCTGTTCTCAAAGTGATTTTGGGAGAA	59	407
				Rev: ACCTGAAGACTGGAGAGTGAGTTAAATGCT		
*TLR9*	EU170539	2848 G > A	External	For: GTCAATGGCTCCCAGTTCC	52	292
		(rs352140)		Rev: CATTGCCGCTGAAGTCCA		
			Internal	For: AAGCTGGACCTCTACCACGA	59	177
				Rev: TTGGCTGTGGATGTTGTT		

^a^ No., number

^b^ SNP, single nucleotide polymorphism

^c^ bps, base pairs

### Statistical analysis

Distribution of genotypes and alleles in the analyzed *TLR2*, *TLR4* and *TLR9* SNPs among HCMV-infected and uninfected pregnant women was estimated by means of descriptive statistics. The studied groups of patients were tested for the Hardy-Weinberg (H-W) equilibrium, the linkage disequilibrium (LD) and haplotypes, using the SNPStats software (http://bioinfo.iconcologia.net/en/SNPStats_web). Genotypes in all the analyzed polymorphisms preserved the H-W equilibrium in pregnant women, both infected with HCMV and control uninfected individuals (*P* > 0.050). The *TLR4* 896 A > G and 1196 C > T SNPs were in linkage disequilibrium among the studied groups of pregnant women (*P* ≤ 0.050). The relationships between the genotypes, alleles or haplotypes in *TLR* SNPs and the occurrence of the viral infections were determined by cross-tabulation, Pearson’s Chi-squared test and by the logistic regression model. The multiple-SNP analysis for the haplotypes in *TLR4* SNPs, as well as for the complex genotypic variants within the range of all the analyzed *TLR* SNPs was performed by the Expectation Maximization (EM) algorithm. The outcomes of the analyses were determined statistically significant when the significance level of *P* ≤ 0.050 was obtained. The statistical analysis was in part supported by the NCSS 2004 software.

## Results

### Prevalence of anti-HCMV IgG and IgM antibodies

In pregnant women, HCMV infection during pregnancy was estimated on the basis of IgG seroconversion within gestation, on the presence of IgG and IgM specific antibodies or on a low IgG avidity index. IgG seropositivity was confirmed in 96.55% (56/58) of the infected pregnant women, while data on IgM antibodies suggestive of the recent infection, were obtained for 86.21% (50/58) of infected individuals. Control group in the study, consisted of pregnant women seronegative for both IgG and IgM antibodies against HCMV, who were classified as uninfected individuals.

### HCMV DNA loads in body fluids

The median load of HCMV DNA in whole blood specimens of the infected pregnant women was 3.8 × 10^2^ copies/ml and ranged from 1.3 × 10^2^ to 1.1 × 10^3^ copies/ml, while the mean viral load was 5.2 × 10^2^ copies/ml. In case of plasma specimens, the median viral load was 5.6 × 10^2^ copies/ml, ranging from 1.8 × 10^2^ to 3.6 × 10^3^ copies/ml, whereas the mean viral load was 1.4 × 10^3^ copies/ml. Regarding urine samples, the median HCMV DNA load was 4.4 × 10^2^ copies/ml, ranging from 1.5 × 10^2^ to 2.4 × 10^3^ copies/ml, and the mean viral load was 3.2 × 10^3^ copies/ml. Considering fetal amniotic fluids, the median HCMV DNA load was 9.9 × 10^2^ copies/ml, and ranged from 2.2 × 10^2^ to 1.5 × 10^3^ copies/ml, while the mean viral load was 9.2 × 10^2^ copies/ml. In umbilical cord blood samples, the median HCMV DNA load was 3.2 × 10^3^ copies/ml, and ranged from 1.6 × 10^2^ to 6.6 × 10^3^ copies/ml, and the mean viral load was 3.3 × 10^3^ copies/ml.

###  Frequencies of the genotypes in *TLR2*, *TLR4* and *TLR9* SNPs

In the pregnant women, infected with HCMV, the frequencies of GG and GA genotypes found at *TLR2* 2258 G > A polymorphic site were 93.9% (62/66) and 6.1% (4/66), respectively (see Table 2). In cases of *TLR4* SNPs, AA and AG genotypes in 896 A > G SNP were carried by 91.8% (56/61) and 8.2% (5/61) of the pregnant women, respectively, while CC and CT genotypes in 1196 C > T polymorphism—by 90.9% (60/66) and 9.1% (6/66), respectively. Considering *TLR9* 2848 G > A SNP, the GG, GA and AA genotypes were determined in 13.3% (8/60), 41.7% (25/60), and 45% (27/60) of patients, respectively. Among uninfected pregnant women, the frequencies of GG and GA genotypes in *TLR2* SNP were 92.2% (59/64) and 7.8% (5/64), respectively. The frequencies of AA and AG genotypes in *TLR4* 896 A > G were 86.9% (53/61) and 13.1% (8/61), respectively, while the rates of CC and CT genotypes in *TLR4* 1196 C > T were 87.3% (55/63) and 12.7% (8/63), respectively. In cases of *TLR9* 2848 G > A polymorphism, the GG, GA and AA genotypes were observed in 20.3% (13/64), 53.1% (34/64), and 26.6% (17/64), respectively. Taking that into account, the GG homozygotic and GA heterozygotic status decreased significantly the occurrence of HCMV infection (OR 0.44, 95% CI 0.21–0.94 in the dominant model; *P* ≤ 0.050; see Table 2). In turn, the frequencies of the genotypes, located in *TLR2* and *TLR4* SNPs, were similar between the studied groups of the infected and uninfected pregnant women. Moreover, both the haplotypes in *TLR4* polymorphisms and the complex variants within the range of all the analyzed SNPs, were observed in similar frequencies among the studied groups of patients. Considering congenital transmission of HCMV from the infected pregnant women to their fetuses, similar frequencies of various genetic variants of the analyzed polymorphisms, were observed between mothers of congenitally infected and uninfected offsprings.

**Table 2:** Relationship between *TLR2*, *TLR4* and *TLR9* SNPs and the occurrence of HCMV infection among pregnant women

Gene polymorphism	Genetic model	Genotype	Genotype prevalence rates; n (%) ^a^		OR^b^ (95% CI) ^c^	*P*-value^d^
			Infected cases	Uninfected controls		
*TLR2* 2258 G > A	-	GG	62 (93.9%)	59 (92.2%)	1.00	
		GA	4 (6.1%)	5 (7.8%)	0.76 (0.19–2.97)	0.690
*TLR4* 896 A > G	-	AA	56 (91.8%)	53 (86.9%)	1.00	
		AG	5 (8.2%)	8 (13.1%)	0.59 (0.18–1.92)	0.380
*TLR4* 1196 C > T	-	CC	60 (90.9%)	55 (87.3%)	1.00	
		CT	6 (9.1%)	8 (12.7%)	0.69 (0.22–2.11)	0.510
*TLR9* 2848 G > A	Codominant	GG	8 (13.3%)	13 (20.3%)	0.39 (0.13–1.13)	0.093
		GA	25 (41.7%)	34 (53.1%)	0.46 (0.21–1.03)	
		AA	27 (45.0%)	17 (26.6%)	1.00	
	Dominant	AA	27 (45.0%)	17 (26.6%)	1.00	
		GA-GG	33 (55.0%)	47 (73.4%)	0.44 (0.21–0.94)	0.032
	Recessive	AA-GA	52 (86.7%)	51 (79.7%)	1.00	
		GG	8 (13.3%)	13 (20.3%)	0.60 (0.23–1.58)	0.300
	Overdominant	GG-AA	35 (58.3%)	30 (46.9%)	1.00	
		GA	25 (41.7%)	34 (53.1%)	0.63 (0.31–1.28)	0.200

^a^ n, number of tested pregnant women

^b^ OR, odds ratio

^c^ 95% CI, confidence interval

^d^ logistic regression model; *P* ≤ 0.050 is considered as significant

### Distribution of the alleles in *TLR2*, *TLR4* and *TLR9* SNPs

In the infected pregnant women, the frequencies of G and A alleles in *TLR2* 2258 G > A SNP were 97.0% (128/132) and 3.0% (4/132), respectively (see Table 3). In case of *TLR4* polymorphisms, A and G alleles in 896 A > G SNP were determined in 96.0% (117/122) and 4.0% (5/122) of the patients, respectively, while C and T alleles in 1196 C > T SNP—in 95.0% (126/132) and 5.0% (6/132), respectively. Regarding *TLR9* 2848 G > A SNP, G and A alleles were carried by 34.0% (41/120) and 66.0% (79/120) of the infected pregnant women, respectively. Among uninfected pregnant women, the frequencies of G and A alleles in *TLR2* 2258 G > A SNP were 96.0% (123/128) and 4.0% (5/128), respectively. The frequencies of A and G alleles in *TLR4* 896 A > G SNP were 93.0% (114/122) and 7.0% (8/122), respectively, and the rates of C and T alleles in *TLR4* 1196 C > T SNP were 94.0% (118/126) and 6.0% (8/126), respectively. Considering *TLR9* SNP, the G and A alleles were found in frequencies of 47.0% (60/128) and 53.0% (68/128), respectively. The G allele in *TLR9* SNP was significantly more frequent among the uninfected pregnant women, as compared to the infected ones (χ^2^ = 4.14, *P* ≤ 0.050; Pearson’s Chi-squared test; see Table 3). The distribution of the alleles at other analyzed polymorphic sites was similar between the studied groups of patients.

**Table 3:** Distribution of the alleles, located in *TLR2*, *TLR4* and *TLR9* SNPs

Gene polymorphism and allele		No.^a^ of carriers with *TLR* alleles (%)		*P*-value^b^
		Cases	Controls	
*TLR2* 2258 G > A				
	G	128 (97%)	123 (96%)	0.699
	A	4 (3%)	5 (4%)	
*TLR4* 896 A > G				
	A	117 (96%)	114 (93%)	0.392
	G	5 (4%)	8 (7%)	
*TLR4* 1196 C > T				
	C	126 (95%)	118 (94%)	0.523
	T	6 (5%)	8 (5%)	
*TLR9* 2848 G > A				
	G	41 (34%)	60 (47%)	0.042
	A	79 (66%)	68 (53%)	

^a^ No. - number

^b^ Pearson’s Chi-squared test; *P* ≤ 0.050 is considered significant

## Discussion

Both GG homozygous and GA heterozygous variants in *TLR9* 2848 G > A SNP were found in our study to have been significantly associated with a decreased occurrence of HCMV infection among pregnant women. Additionally, the G allele in *TLR9* SNP was observed significantly more frequently among the uninfected pregnant women than among the infected ones. The frequencies of the genotypes and alleles within *TLR9* 2848 G > A SNP determined among uninfected pregnant women, but not among HCMV-infected patients, were similar to the frequencies reported for European populations (see https://www.ncbi.nlm.nih.gov/projects/SNP/snp_ref.cgi?rs=352140).

Differences in distribution of both the genotypes and alleles within *TLR9* 2848 G > A SNP between the infected pregnant women analyzed in the current study, and the reported European populations might be due to a selection bias. Similarly, significantly higher prevalence rates of anti-HCMV IgG and IgM antibodies determined among the infected pregnant women studied in this paper, as compared to the previously reported cohort of Polish pregnant women between 2010 and 2011, as well as to other European populations, are possibly also caused by a classification of the patients to the presented study [4–7]. So far, it has been the first study to reveal a really significant role of *TLR9* 2848 G > A SNP in the occurrence of HCMV infection in pregnant women. Another research reported before that the GA heterozygotic status in the analyzed *TLR9* SNP, carried by fetuses and neonates, congenitally infected with HCMV, had significantly increased the risk of the infection [18]. In turn, the G allele in *TLR9* SNP was more frequent among the infected offsprings than among the uninfected ones, although that difference was statistically non-significant [18]. The distinct contribution of the analyzed *TLR9* SNP in the occurrence of HCMV infection may have been age-related, as well as may have resulted from the classification bias. The qualification criteria of the pregnant women to the study included their serological status, the presence of HCMV DNA in their body fluids and were also based on confirmed congenital infection with the virus in their fetuses. In turn, the fetuses were diagnosed as congenitally infected with HCMV only on the basis of ultrasound markers and on the presence of HCMV DNA in their body fluids, since the diagnostics towards the infection is not routinely performed in pregnant women. Considering the role of *TLR9* SNP in HCMV infection, the TT homozygotic status in *TLR9* -1237 T > C SNP was reported to have been correlated with a decreased risk of the infection in HCMV-seropositive kidney transplant recipients [50]. In recipients of allogeneic hematopoietic stem cell transplants, the HCMV infection occurred significantly more frequently among carriers of minor C allele in *TLR9* -1237 T > C, as compared to the patients, carrying the T allele [51]. On the other hand, the *TLR9* 2848 G > A polymorphism was not involved in the incidence of HCMV infection among the examined patients [51]. However, *TLR9* -1486 T > C and 2848 C > T SNPs were associated with an increased risk of HCMV disease among infants [33]. Therefore, it seems possible that different SNPs, residing within *TLR9* gene, may have been correlated with various disease types. Regarding the HCMV infection, previous papers showed some contribution of TLR9 to its occurrence [14, 22, 23]. In case of plasmacytoid dendritic cells (pDCs), the infection with HCMV was significantly associated with increased levels of TLR9 [52]. The inhibited cytokine expression, observed after treatment of pDCs with CpG, agonists for TLR7 and TLR9, suggested an involvement of the reported TLRs in the development of HCMV infection [52]. What is more, a significant role in the occurrence of HCMV infection was previously confirmed for TLR2 and TLR4 molecules as well [30, 53, 54]. TLR2 was found to have been involved in the functional sensing of HCMV through a direct interaction with the viral glycoproteins gB and gH [20]. In case of TLR4, the molecule was involved in an inhibition of HCMV infection [21]. The expression levels of both TLR2 and TLR4 were correlated with the levels of HCMV IE1-72 protein [55]. Considering genetic alterations in the current study, no differences were found in the distribution of distinct genotypes within the range of both *TLR2* and *TLR4* SNPs. The frequencies of the genotypes in the analyzed *TLR2* and *TLR4* polymorphic sites, found among HCMV-infected pregnant women were similar to the frequencies observed among European populations (see https://www.ncbi.nlm.nih.gov/projects/SNP/snp_ref.cgi?rs=5743708; https://www.ncbi.nlm.nih.gov/projects/SNP/snp_ref.cgi?rs=4986790; https://www.ncbi.nlm.nih.gov/projects/SNP/snp_ref.cgi?rs=4986791). Taking into account the allelic variability within *TLR2* 2258 G > A and *TLR4* 1196 C > T SNPs, the frequencies observed among all pregnant women studied in our research, were similar to the frequencies reported for European populations. In case of *TLR4* 896 C > T SNP, similarity to the European populations was found for HCMV-infected pregnant women (see https://www.ncbi.nlm.nih.gov/projects/SNP/snp_ref.cgi?rs=4986790). So far, no other study has shown before any possible involvement of *TLR2* 2258 G > A, *TLR4* 896 A > G and *TLR4* 1196 C > T SNPs in the occurrence of HCMV infection during pregnancy. However, in case of congenital HCMV infection, GA heterozygotic status and A allele within *TLR2* 2258 G > A, as well as CC genotype in the other *TLR2* + 1350 T > C SNP, was reported to be correlated with the infection [27, 28]. Regarding presented data, the age-dependent type of the involvement of *TLR2* 2258 G > A polymorphism to HCMV infection seems to be quite possible. Considering *TLR4* SNPs, the CC genotype in *TLR4* 1196 C > T SNP, GC haplotype in both analyzed *TLR4* SNPs, as well as GCA multiple variants within the range of *TLR4* and *TLR9* 2848 G > A SNPs, were found to have been correlated with congenital HCMV infection in fetuses and neonates [18]. In pregnant women, the frequencies of distinct genotypes in *TLR4* SNPs were similar to those, determined among congenitally infected fetuses and neonates, although no CT genotype at *TLR4* 1196 C > T polymorphic site was observed among the infected offsprings [18]. Among pregnant women, the low genotypic variability within *TLR2* and *TLR4* SNPs seems to be the important cause of the lack of any associations with HCMV infection. Similarly to our study, almost the same frequencies of distinct genotypes in both *TLR2* 2258 G > A and *TLR4* 896 G > A SNPs were determined in patients with transplants, with and without clinical signs of HCMV infection [50]. Regarding the outcomes presented in this report, *TLR9* 2848 G > A SNP seems to be the major polymorphism, contributing to HCMV infection in pregnant women during pregnancy. However, the studied genetic alteration is not involved, either in amino acid changes in TLR9 molecule or in regulatory site modifications within *TLR9* gene [56]. Therefore, other molecular changes occurring simultaneously with the analyzed polymorphism, may contribute to the course of immune response after the infection with HCMV.

## Conclusions

The outcomes presented in the current study show that *TLR9* 2848 G > A SNP seems to be involved in the occurrence of HCMV infection in pregnant women. Since both the GG homozygotic and GA heterozygotic statuses, as well as the G allele at *TLR9* polymorphic site were significantly more frequent among the uninfected pregnant women, when compared with the infected ones, the genetic alterations in the studied polymorphism may plausibly be associated with the infection with HCMV. However, we suggest that further detailed studies would highly be justified to investigate the molecular mechanism of the *TLR9* 2848 G > A SNP function in HCMV infection.

## Additional files

Additional file 1: Figure S1. Exemplary PCR-RFLP products representative for various genotypes within *TLR2* 2258 G > A (A), *TLR4* 896 A > G (B), *TLR4* 1196 C > T (C) and *TLR9* 2848 G > A (D) SNPs. DNA fragments were resolved in 2% agarose gel, stained with ethidium bromide. Disparate lanes show restriction profiles for distinct genotypes in the range of studied *TLR* polymorphisms, determined in different pregnant women. The numbers on the right side of electropherograms show the size of separated DNA fragments. M—50 bp DNA marker; GG, GA, AA, AG, CT, CC—genotypes determined in studied *TLR* polymorphisms. (TIF 501 kb)

Additional file 2: Figure S2. a and b Representative chromatograms of DNA fragments of various sequenced PCR products, encompassing *TLR2* 2258 G > A (A, B), *TLR4* 896 A > G (C, D), *TLR4* 1196 C > T (E, F) and *TLR9* 2848 G > A (G-I) SNPs. For *TLR2* SNP, DNA forward strands were sequenced, and for *TLR4* and *TLR9*—reverse strands were assayed. The numbers above some peaks of chromatograms indicate the following nucleotides determined in sequenced DNA fragments. Loci of the polymorphisms and genotypes analyzed in the study, are indicated with arrows. GG, GA, AA, AG, CT, CC—genotypes determined in studied *TLR* SNPs. (ZIP 1732 kb)
