# Supplementary figures and images for: Toll-like receptors genes polymorphisms and the occurrence of HCMV infection among pregnant women
Source: Virol J. 2017 Mar 24;14:64. doi: 10.1186/s12985-017-0730-8 (PMC5364709; doi:10.1186/s12985-017-0730-8)

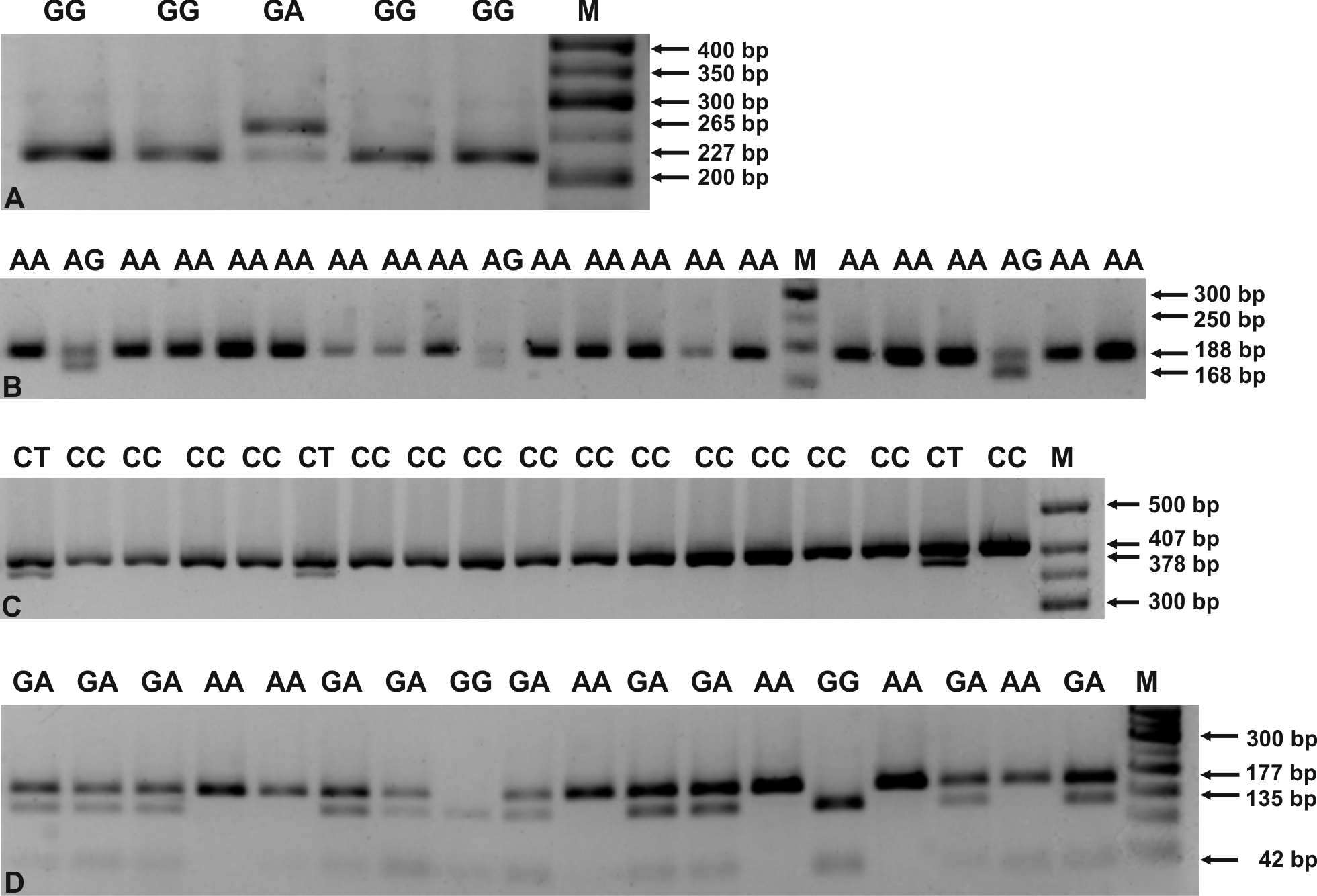

Supplement: Supplementary file 1 — Exemplary PCR-RFLP products representative for various genotypes within TLR2 2258 G > A (A), TLR4 896 A > G (B), TLR4 1196 C > T (C) and TLR9 2848 G > A (D) SNPs. DNA fragments were resolved in 2% agarose gel, stained with ethidium bromide. Disparate lanes show restriction profiles for distinct genotypes in the range of studied TLR polymorphisms, determined in different pregnant women. The numbers on the right side of electropherograms show the size of separated DNA fragments. M—50 bp DNA marker; GG, GA, AA, AG, CT, CC—genotypes determined in studied TLR polymorphisms. (TIF 501 kb) [file 12985_2017_730_MOESM1_ESM.tif]

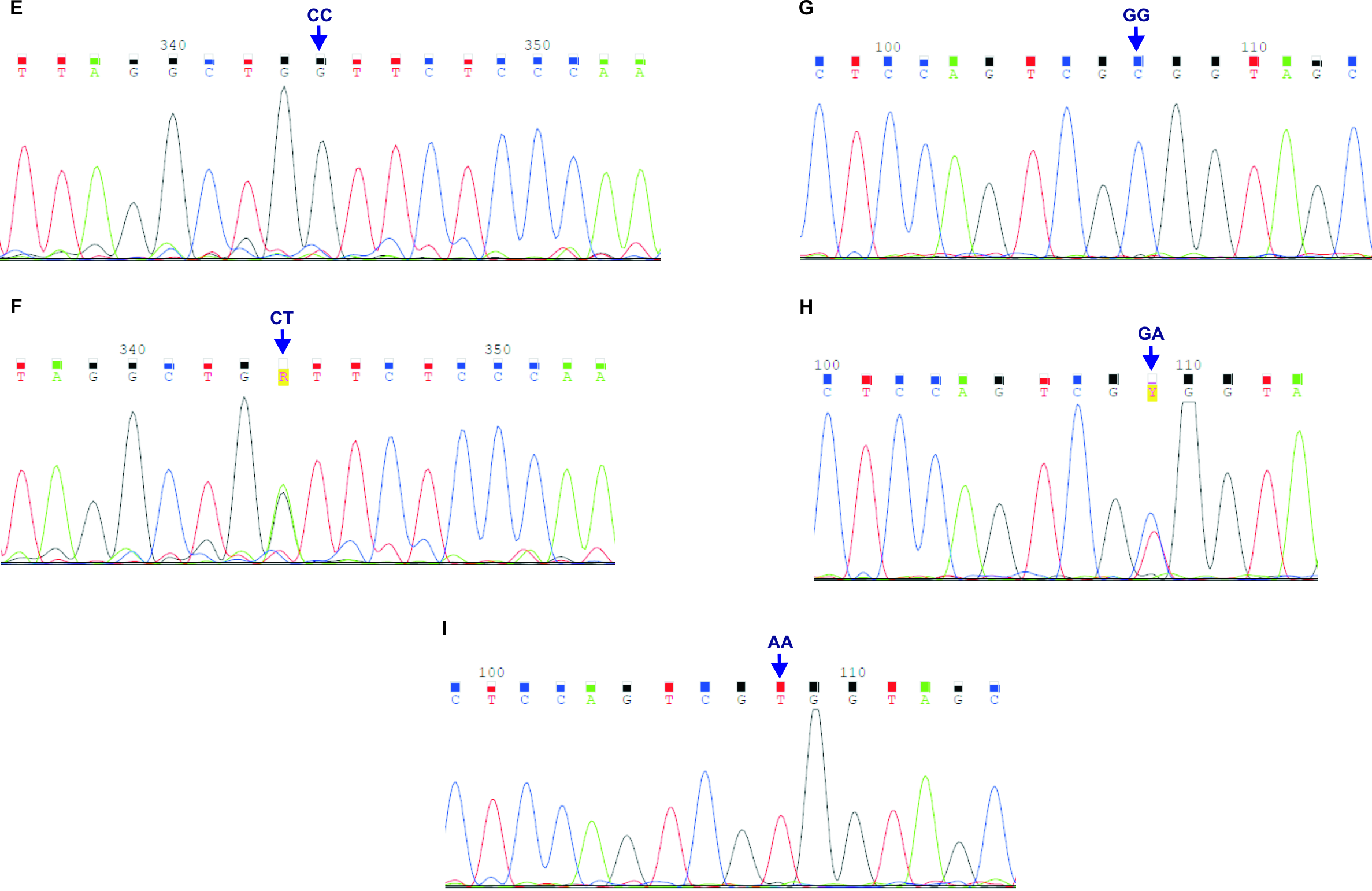

Supplement: Supplementary file 2 — a and b Representative chromatograms of DNA fragments of various sequenced PCR products, encompassing TLR2 2258 G > A (A, B), TLR4 896 A > G (C, D), TLR4 1196 C > T (E, F) and TLR9 2848 G > A (G-I) SNPs. For TLR2 SNP, DNA forward strands were sequenced, and for TLR4 and TLR9—reverse strands were assayed. The numbers above some peaks of chromatograms indicate the following nucleotides determined in sequenced DNA fragments. Loci of the polymorphisms and genotypes analyzed in the study, are indicated with arrows. GG, GA, AA, AG, CT, CC—genotypes determined in studied TLR SNPs. (ZIP 1732 kb) [file 12985_2017_730_MOESM2_ESM.zip › Figure S2b.tif]

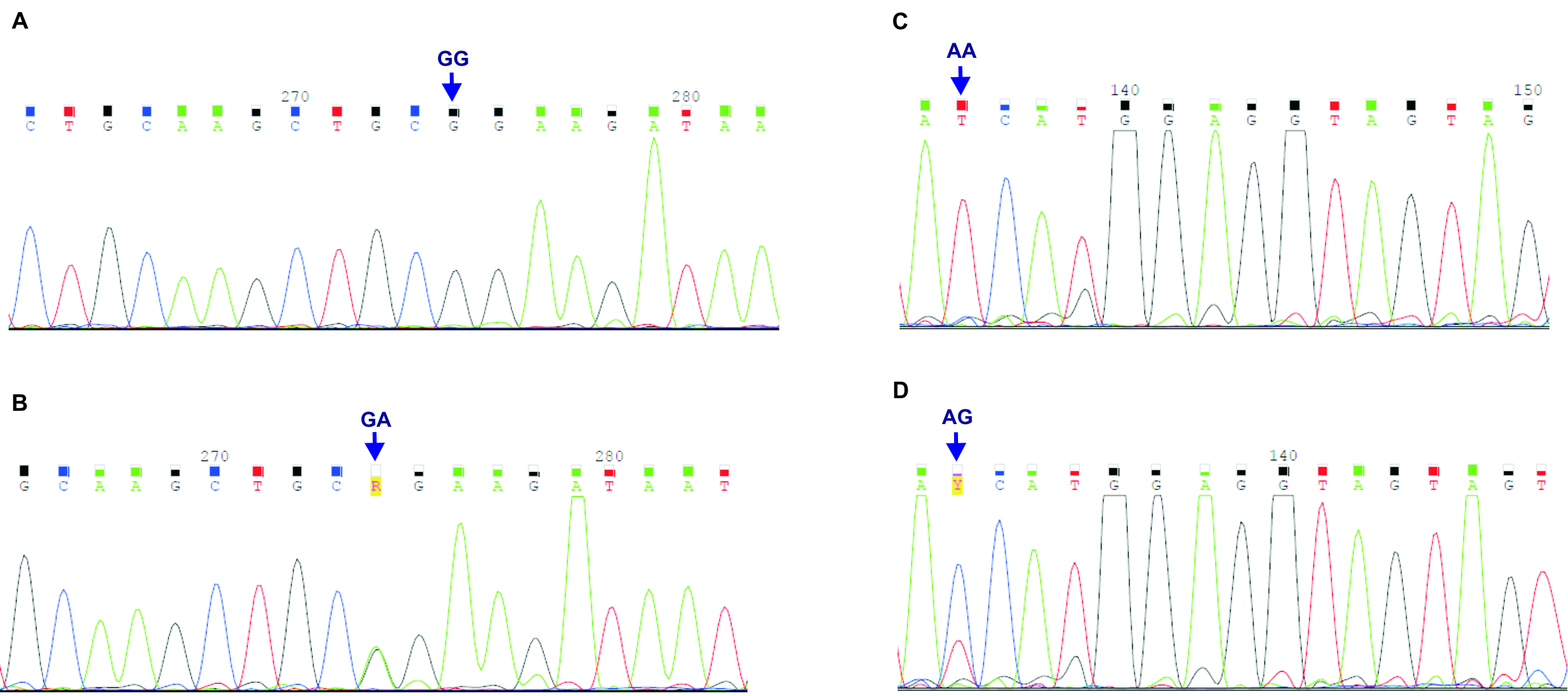

Supplement: Supplementary file 2 — a and b Representative chromatograms of DNA fragments of various sequenced PCR products, encompassing TLR2 2258 G > A (A, B), TLR4 896 A > G (C, D), TLR4 1196 C > T (E, F) and TLR9 2848 G > A (G-I) SNPs. For TLR2 SNP, DNA forward strands were sequenced, and for TLR4 and TLR9—reverse strands were assayed. The numbers above some peaks of chromatograms indicate the following nucleotides determined in sequenced DNA fragments. Loci of the polymorphisms and genotypes analyzed in the study, are indicated with arrows. GG, GA, AA, AG, CT, CC—genotypes determined in studied TLR SNPs. (ZIP 1732 kb) [file 12985_2017_730_MOESM2_ESM.zip › Figure S2a.tif]
